# Synthesis and crystal structure of di­chlorido­(1,10-phenanthroline-κ^2^ *N*,*N*′)gold(III) hexa­fluorido­phosphate

**DOI:** 10.1107/S2056989017008763

**Published:** 2017-06-16

**Authors:** Raphael Enoque Ferraz de Paiva, Douglas Hideki Nakahata, Pedro Paulo Corbi

**Affiliations:** a Institute of Chemistry, University of Campinas - UNICAMP, Campinas - SP, Brazil

**Keywords:** gold(III), 1,10-phenanthroline ligand, square-planar coordination, anion-π inter­actions, crystal structure

## Abstract

A gold(III) salt of composition [AuCl_2_(C_12_H_8_N_2_)]PF_6_ was prepared and characterized by elemental and mass spectrometric analysis (ESI(+)–QTOF–MS), ^1^H nuclear magnetic resonance measurements and by single-crystal X-ray diffraction. The square-planar coordination sphere of Au^III^ comprises the bidentate 1,10-phenanthroline ligand and two chloride ions, with the Au^III^ ion only slightly shifted from the least-squares plane of the ligating atoms (r.m.s. = 0.018 Å). In contrast to two other previously reported Au^III^-phenantroline structures that are stabilized by inter­actions involving the chlorido ligands, the packing of the title compound does not present these features. Instead, the hexa­fluorido­phosphate counter-ion gives rise to anion⋯π inter­actions that are a crucial factor for the crystal packing.

## Abstract 2

The gold(III) atom in the title complex has a square-planar coordination environment defined by two Cl atoms and a chelating phenanthroline ligand.

## Chemical context   

Au^III^ is isoelectronic with Pt^II^ and forms compounds with similar coordination modes and structures. Therefore, the synthesis of Au^III^-based compounds has attracted much inter­est in the field of bioinorganic and medicinal chemistry after the successful application of cis-platin [*cis*-diamminedi­chlorido­platinum(II)] for cancer treatment (Siddik, 2003 ▸). Aromatic N-donors, such as 1,10-phenanthroline, are of inter­est given their planar structure that synergizes well with the typical square-planar coordination sphere of Au^III^, producing potent DNA-inter­calating agents (Abbate *et al.*, 2000 ▸; Zou *et al.*, 2015 ▸). On the other hand, Au^III^ compounds differ from Pt^II^ compounds in terms of their inter­actions with biomolecules, their stability in biological media or their mechanism of action. A review on cytotoxic properties and mechanisms of Au^III^ compounds with N-donors has been provided by Zou *et al.* (2015 ▸).
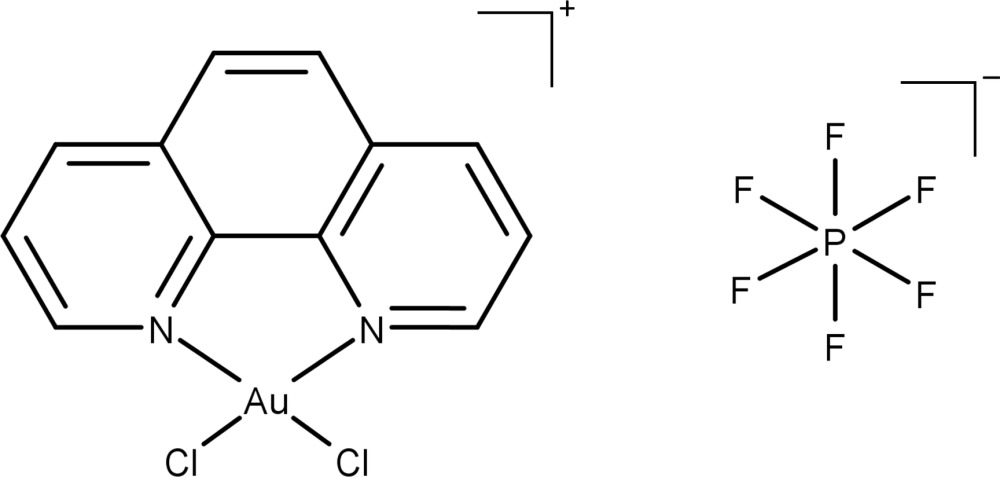


In this context we have prepared the title salt, [AuCl_2_(C_12_H_8_N_2_)]PF_6_, that was characterized by elemental and mass spectrometric analysis (ESI(+)–QTOF–MS), ^1^H nuclear magnetic resonance measurements and by single crystal X-ray diffraction.

## Structural commentary   

**Figure 1:**
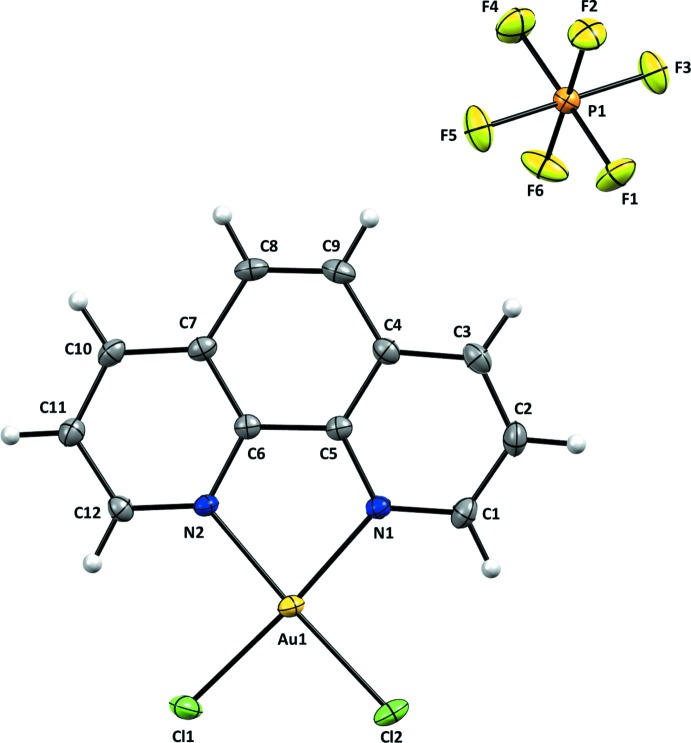
The mol­ecular entities of the title salt [AuCl_2_(C_12_H_8_N_2_)]PF_6_. Displacement ellipsoids are drawn at the 40% probability level. Hydrogen atoms are not labelled for clarity.

All atoms in the title salt are on general positions. The Au^III^ atom has a square-planar coordination environment, with the chlorido ligands in a *cis* configuration to each other. The Au^III^ atom deviates from planarity (as determined based on the four coordinating atoms) by 0.018 Å (r.m.s.). The main bond lengths [Au—N1 = 2.032 (2), Au—N2 = 2.036 (2), Au—Cl1 = 2.251 (1) and Au—Cl2 = 2.255 (1) Å] are in the normal ranges for this kind of complexes (see *Database survey*). The bite angle of the 1,10-phenanthroline ligand is 81.75 (9)°, while the Cl1—Au—Cl2 angle is 89.28 (3)°. Despite the highly symmetrical nature of the hexa­fluorido­phosphate counter-ion, this unit does not show any disorder. The structures of the mol­ecular entities of the [AuCl_2_(C_12_H_8_N_2_)]PF_6_ salt are shown in Fig. 1 ▸.

## Supra­molecular features   

**Figure 2:**
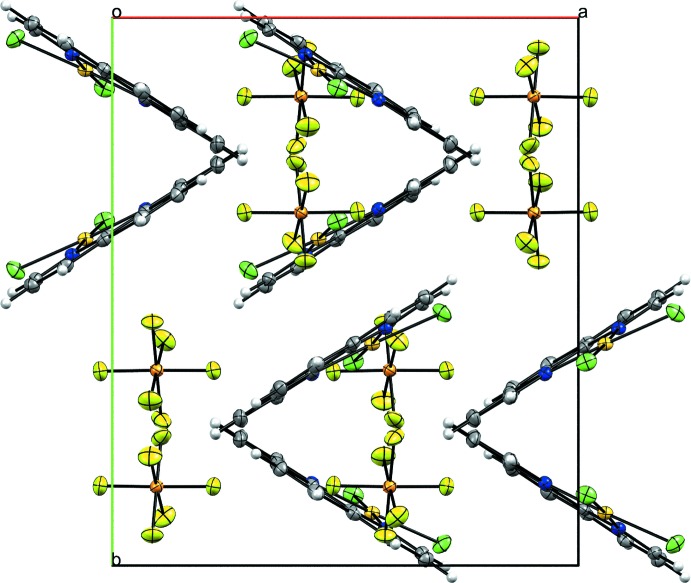
Packing of the crystal structure of [AuCl_2_(C_12_H_8_N_2_)]PF_6_ in a view along the *c* axis. Displacement ellipsoids are drawn at the 40% probability level.

**Figure 3:**
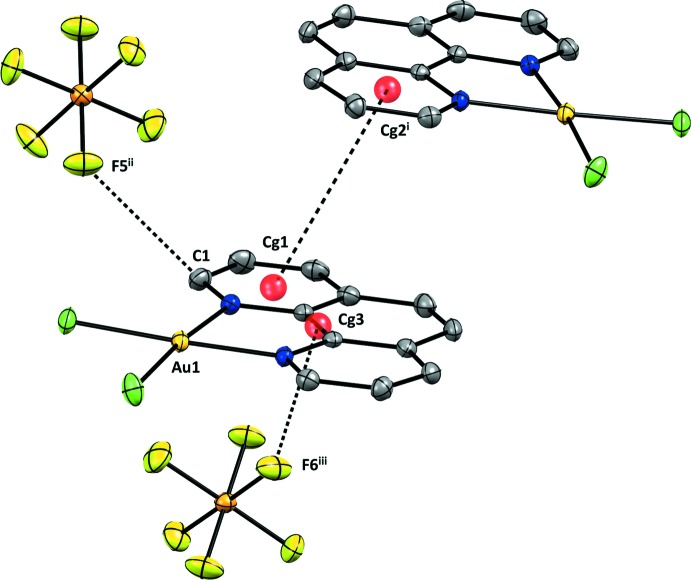
Inter­molecular inter­actions present in the crystal structure. Displacement ellipsoids are drawn at the 40% probability level. Hydrogen atoms were omitted for clarity. [Symmetry codes: (i) 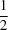 + *x*, *y*, 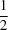 − *z*, (ii) *x*, 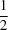 − *y*, 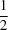 + *z*, (iii) −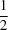 + *x*, *y*, 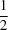 − *z*.]

The mol­ecular packing in the crystal is shown in Fig. 2 ▸. Despite the square-planar coordination environment around Au^III^ and the presence of the highly conjugated and planar 1,10-phenanthroline ligand, π–π inter­actions have little relevance to the stabilization of the crystal. The shortest π-like inter­action between the centroids [*Cg*1⋯*Cg*2^i^; symmetry code: (i) 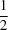 + *x*, *y*, 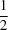 − *z*; Fig. 3 ▸] of two neighbouring 1,10-phenanthroline rings are associated with a distance of 4.2521 (15) Å, which is very close to the upper limit of the threshold established by Janiak (2000 ▸) for a relevant offset π inter­action.

The inter­actions between the hexa­fluorido­phosphate counter-ion and the 1,10-phenanthroline ligands constitute the major inter­molecular inter­actions in the crystal and can be divided into two types. The first type corresponds to an anion-donor⋯ π-acceptor inter­action (Chifotides & Dunbar, 2013 ▸), with the shortest contact being C1⋯F5^ii^, of 3.096 (4) Å [symmetry code: (ii) *x*, ½ − *y*, ½ + *z*; Fig. 3 ▸]. The second and unique type of inter­action between the PF_6_^−^ anion and the π system of the phenanthroline ligand is observed where fluorine atoms point directly to the mid-point of an aromatic C—C bond. The distance between F6^ii^ and the mid-point of C5 and C6 is 2.822 Å. The individual distances are C5⋯F6^ii^ 2.925 (3) and C6⋯F6^iii^ 2.894 (3) Å [symmetry code: (iii) −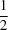 + *x*, *y*, 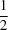 − *z*].

## Database survey   

A few structures of Au^III^-(1,10-phenanthroline) compounds have been reported in the literature with different counter-ions. Abbate *et al.* (2000 ▸) reported the monohydrate chloride structure that crystallizes in the space group type *P*2_1_/*n*, with Au—N distances of 2.033 (8) and 2.056 (8) Å and Au—Cl distances of 2.266 (3) and 2.263 (3) Å, respectively. The N—Au—N angle is 82.0 (3)° and the Cl—Au—Cl angle 89.5 (1)°. Pitteri *et al.* (2008 ▸) determined the structure with a disordered [AuBrCl(CN)_2_]^−^ unit as a counter-ion in space group type *P* 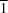. The Au—N distances are 2.05 (1) and 2.05 (1) Å, while the Au—Cl distances are 2.290 (5) and 2.299 (5) Å. The title compound has Au—N distances similar to that of the structure reported by Abbate *et al.* (2000 ▸), but slightly shorter than the one by Pitteri *et al.* (2008 ▸). Regarding the Au—Cl distances, [AuCl_2_(C_12_H_8_N_2_)]PF_6_ and the structure reported by Abbate *et al.* (2000 ▸) have shorter ones than that reported by Pitteri *et al.* (2008 ▸). Although the [AuCl_2_(C_12_H_8_N_2_)]^+^ cations in the three structures exhibit no significant differences, their crystal packings vary greatly as a consequence of the inter­molecular inter­actions with the different counter-ions. The structure reported by Abbate *et al.* (2000 ▸) has the Au^III^-(1,10-phenanthroline) units closer in space, with the shortest centroid-to-centroid distance being 3.820 Å, much closer than 4.2521 (15) Å observed in the title compound. Furthermore, the presence of a water mol­ecule and the chloride counter-ion establish a classical hydrogen-bonding network, which is absent in the structure of the title compound. The structure determined by Pitteri *et al.* (2008 ▸) is the only one with an axial Au⋯*L* inter­action, namely Au⋯Br (3.374 Å).

## Synthesis and crystallization   

[AuCl_2_(C_12_H_8_N_2_)]PF_6_ was synthesized by a modification of a literature protocol (Casini *et al.*, 2010 ▸): K[AuCl_4_] (0.25 mmol, 95.0 mg) was dissolved in 3 ml of H_2_O/CH_3_CN (1:5, *v*/*v*), and 1,10-phenanthroline, (0.25 mmol, 45 mg) dissolved in 0.5 ml of CH_3_CN was then added to the gold(III)-containing solution. Finally, NH_4_PF_6_ (0.75 mmol, 124.6 mg) was added to the solution and the mixture was refluxed for 16 h. The obtained solid was isolated by filtration, washed with cold water and dried *in vacuo*. Elemental Analysis was performed on an Elemental Analyzer CHNS-O 2400 Perkin Elmer. Anal. Calcd. for C_12_H_8_AuCl_2_F_6_N_2_P (593.04 g mol^−1^): C 24.30%, H 1.36%, N 4.72%. Found: C 24.08%, H 0.70%, N 4.73%. Mass spectra were acquired in a XEVO QTOF–MS instrument (Waters). The sample was dissolved in the smallest possible volume of DMSO and diluted in a 1:1 (*v*/*v*) mixture of water and aceto­nitrile containing 0.1% formic acid. ESI(+)–QTOF–MS (*m*/*z*, [AuCl_2_(C_12_H_8_N_2_)]^+^, 100% relative abundance): 446. 9707 (calculated 446.9730). Crystals suitable for single crystal X-ray analysis were obtained by recrystallization from aceto­nitrile solution.

## Solution stability   

**Figure 4:**
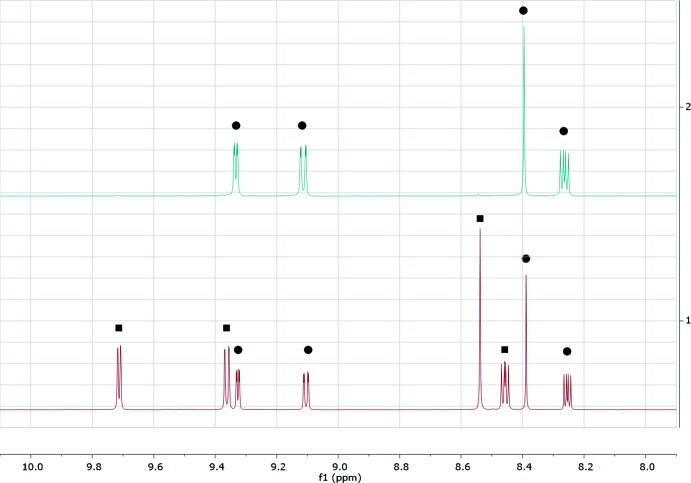
^1^H NMR spectra following the Cl replacement by DMSO-*d*_6_ in the salt [Au(phen)Cl_2_]PF_6_, where phen = 1,10-phenanthroline. (Top) Spectrum obtained from a freshly dissolved sample and (bottom) 72 h after dissolution. Two populations were identified, [Au(phen)Cl_2_]^+^ (symbolized by a black square) and a chloride replacement product, most likely [Au(phen)(dmso-*d*_6_)_2_]^3+^ (symbolized by a black dot).

The stability of the [Au(1,10-phenanthroline)]^3+^ moiety is critical for the biological properties of the compound, including cytotoxicity. The [AuCl_2_(C_12_H_8_N_2_)]PF_6_ salt was dissolved in deuterated di­methyl­sulfoxide (DMSO-*d*6) and the solvent replacement was followed by ^1^H NMR for 72 h (Fig. 4 ▸). ^1^H NMR spectra were acquired on a Bruker Avance III 400 MHz. The labile chlorido ligands were replaced, as expected, but the [Au(1,10-phenanthroline)]^3+^ moiety remained stable in the presence of the coordinating solvent (DMSO) throughout the period evaluated.

## Refinement details   

Crystal data, data collection and structure refinement details are summarized in Table 1 ▸. H atoms were set in calculated positions, with C—H = 0.95 Å and *U*_iso_(H) = 1.2*U*_eq_(C).

## Supplementary Material

Crystal structure: contains datablock(s) I. DOI: 10.1107/S2056989017008763/wm5398sup1.cif

Structure factors: contains datablock(s) I. DOI: 10.1107/S2056989017008763/wm5398Isup2.hkl

Supporting information file. DOI: 10.1107/S2056989017008763/wm5398Isup3.mol

CCDC reference: 1555623

Additional supporting information:  crystallographic information; 3D view; checkCIF report

## Figures and Tables

**Table 1:** Experimental details

Crystal data	
Chemical formula	[AuCl_2_(C_12_H_8_N_2_)]PF_6_
*M*_r_	593.04
Crystal system, space group	Orthorhombic, *P* *b* *c* *a*
Temperature (K)	150
*a*, *b*, *c* (Å)	12.9983 (7), 15.2709 (10), 15.5153 (10)
*V* (Å^3^)	3079.7 (3)
*Z*	8
Radiation type	Mo *K*α
μ (mm^−1^)	10.07
Crystal size (mm)	0.15 × 0.13 × 0.05

Data collection	
Diffractometer	Bruker APEX CCD detector
Absorption correction	Multi-scan (*SADABS*; Bruker, 2010 ▸)
*T*_min_, *T*_max_	0.576, 0.746
No. of measured, independent and observed [*I* > 2σ(*I*)] reflections	15573, 3822, 3192
*R*_int_	0.027
(sin θ/λ)_max_ (Å^−1^)	0.667

Refinement	
*R*[*F* ^2^ > 2σ(*F* ^2^)], *wR*(*F* ^2^), *S*	0.019, 0.040, 1.01
No. of reflections	3822
No. of parameters	217
H-atom treatment	H-atom parameters constrained
Δρ_max_, Δρ_min_ (e Å^−3^)	1.08, −0.56

Computer programs: *APEX2* and *SAINT* (Bruker, 2010 ▸), *SHELXS97* (Sheldrick, 2008 ▸), *SHELXL2014* (Sheldrick, 2015 ▸), *Mercury* (Macrae *et al.*, 2006 ▸) and *publCIF* (Westrip, 2010 ▸).

## supplementary crystallographic information

### Crystal data

**Table:**

[AuCl_2_(C_12_H_8_N_2_)]PF_6_	*D*_x_ = 2.558 Mg m^−^^3^
*M**_r_* = 593.04	Mo *K*α radiation, λ = 0.71073 Å
Orthorhombic, *P**b**c**a*	Cell parameters from 119 reflections
*a* = 12.9983 (7) Å	θ = 3.4–27.3°
*b* = 15.2709 (10) Å	µ = 10.07 mm^−^^1^
*c* = 15.5153 (10) Å	*T* = 150 K
*V* = 3079.7 (3) Å^3^	Plate, yellow
*Z* = 8	0.15 × 0.13 × 0.05 mm
*F*(000) = 2208	

### Data collection

**Table:**

Bruker APEX CCD detector diffractometer	3822 independent reflections
Radiation source: fine-focus sealed tube	3192 reflections with *I* > 2σ(*I*)
Detector resolution: 8.3333 pixels mm^-1^	*R*_int_ = 0.027
phi and ω scans	θ_max_ = 28.3°, θ_min_ = 2.4°
Absorption correction: multi-scan (*SADABS*; Bruker, 2010)	*h* = −17→15
*T*_min_ = 0.576, *T*_max_ = 0.746	*k* = −20→15
15573 measured reflections	*l* = −15→20

### Refinement

**Table:**

Refinement on *F*^2^	0 restraints
Least-squares matrix: full	Hydrogen site location: inferred from neighbouring sites
*R*[*F*^2^ > 2σ(*F*^2^)] = 0.019	H-atom parameters constrained
*wR*(*F*^2^) = 0.040	*w* = 1/[σ^2^(*F*_o_^2^) + (0.0194*P*)^2^] where *P* = (*F*_o_^2^ + 2*F*_c_^2^)/3
*S* = 1.01	(Δ/σ)_max_ = 0.001
3822 reflections	Δρ_max_ = 1.08 e Å^−^^3^
217 parameters	Δρ_min_ = −0.56 e Å^−^^3^

### Special details

**Table:**

Geometry. All esds (except the esd in the dihedral angle between two l.s. planes) are estimated using the full covariance matrix. The cell esds are taken into account individually in the estimation of esds in distances, angles and torsion angles; correlations between esds in cell parameters are only used when they are defined by crystal symmetry. An approximate (isotropic) treatment of cell esds is used for estimating esds involving l.s. planes.

### Fractional atomic coordinates and isotropic or equivalent isotropic displacement parameters (Å^2^)

**Table:**

	*x*	*y*	*z*	*U*_iso_*/*U*_eq_	
Au1	0.44227 (2)	0.09568 (2)	0.04214 (2)	0.01802 (4)	
Cl1	0.29239 (6)	0.03883 (5)	−0.00417 (5)	0.03186 (17)	
Cl2	0.48300 (6)	0.12866 (5)	−0.09535 (5)	0.02952 (17)	
N1	0.57092 (15)	0.15008 (15)	0.09387 (16)	0.0184 (5)	
N2	0.41395 (16)	0.06735 (15)	0.16813 (15)	0.0170 (5)	
C1	0.6465 (2)	0.1908 (2)	0.0529 (2)	0.0254 (7)	
H1	0.6468	0.1921	−0.0083	0.030*	
C2	0.7252 (2)	0.2317 (2)	0.0982 (2)	0.0295 (7)	
H2	0.7786	0.2608	0.0677	0.035*	
C3	0.7262 (2)	0.2303 (2)	0.1863 (2)	0.0270 (7)	
H3	0.7794	0.2591	0.2172	0.032*	
C4	0.6474 (2)	0.18577 (19)	0.2310 (2)	0.0221 (6)	
C5	0.57038 (18)	0.14663 (18)	0.18156 (19)	0.0173 (6)	
C6	0.48791 (19)	0.10177 (17)	0.22131 (18)	0.0166 (5)	
C7	0.4824 (2)	0.09379 (18)	0.31033 (19)	0.0204 (6)	
C8	0.5628 (2)	0.1336 (2)	0.3608 (2)	0.0241 (6)	
H8	0.5611	0.1288	0.4218	0.029*	
C9	0.6403 (2)	0.1775 (2)	0.3226 (2)	0.0260 (7)	
H9	0.6918	0.2036	0.3576	0.031*	
C10	0.3993 (2)	0.04620 (19)	0.3444 (2)	0.0230 (6)	
H10	0.3929	0.0386	0.4050	0.028*	
C11	0.3274 (2)	0.01083 (19)	0.29003 (19)	0.0240 (6)	
H11	0.2715	−0.0218	0.3129	0.029*	
C12	0.3362 (2)	0.02256 (17)	0.20087 (19)	0.0210 (6)	
H12	0.2858	−0.0019	0.1636	0.025*	
P1	0.40487 (5)	0.14377 (5)	0.61776 (5)	0.01992 (16)	
F1	0.38930 (15)	0.08895 (13)	0.70331 (12)	0.0412 (5)	
F2	0.41427 (15)	0.05546 (12)	0.56412 (13)	0.0362 (5)	
F3	0.52589 (12)	0.14548 (12)	0.63152 (14)	0.0399 (5)	
F4	0.41879 (16)	0.19932 (14)	0.53096 (13)	0.0443 (5)	
F5	0.28305 (12)	0.14421 (12)	0.60406 (14)	0.0410 (5)	
F6	0.39496 (14)	0.23393 (12)	0.67051 (14)	0.0423 (5)	

### Atomic displacement parameters (Å^2^)

**Table:**

	*U*^11^	*U*^22^	*U*^33^	*U*^12^	*U*^13^	*U*^23^
Au1	0.02348 (6)	0.01722 (6)	0.01336 (6)	0.00051 (4)	−0.00115 (4)	−0.00098 (4)
Cl1	0.0364 (4)	0.0344 (4)	0.0249 (4)	−0.0106 (3)	−0.0115 (3)	0.0001 (4)
Cl2	0.0419 (4)	0.0329 (4)	0.0138 (3)	0.0045 (3)	0.0028 (3)	−0.0002 (3)
N1	0.0192 (11)	0.0202 (12)	0.0159 (12)	0.0014 (9)	0.0006 (9)	−0.0011 (10)
N2	0.0210 (11)	0.0166 (11)	0.0134 (12)	0.0001 (9)	−0.0008 (10)	−0.0012 (10)
C1	0.0246 (14)	0.0276 (16)	0.0239 (17)	0.0031 (12)	0.0071 (13)	0.0020 (13)
C2	0.0211 (14)	0.0331 (18)	0.0342 (19)	−0.0027 (13)	0.0067 (13)	0.0066 (15)
C3	0.0183 (14)	0.0269 (16)	0.0359 (19)	0.0008 (12)	−0.0035 (13)	−0.0017 (14)
C4	0.0189 (13)	0.0219 (15)	0.0256 (16)	0.0023 (11)	−0.0023 (12)	−0.0007 (13)
C5	0.0178 (13)	0.0168 (13)	0.0173 (14)	0.0044 (10)	−0.0005 (11)	−0.0010 (11)
C6	0.0177 (12)	0.0140 (12)	0.0182 (14)	0.0043 (10)	−0.0007 (11)	−0.0005 (12)
C7	0.0236 (13)	0.0192 (13)	0.0184 (15)	0.0051 (11)	−0.0007 (12)	0.0011 (12)
C8	0.0304 (15)	0.0259 (15)	0.0158 (15)	0.0058 (12)	−0.0056 (13)	−0.0020 (13)
C9	0.0252 (15)	0.0277 (16)	0.0250 (17)	0.0017 (12)	−0.0082 (13)	−0.0068 (14)
C10	0.0266 (14)	0.0237 (15)	0.0187 (15)	0.0048 (12)	0.0022 (13)	0.0040 (13)
C11	0.0261 (14)	0.0233 (14)	0.0227 (16)	−0.0014 (12)	0.0034 (13)	0.0020 (13)
C12	0.0208 (13)	0.0178 (13)	0.0244 (16)	−0.0014 (11)	−0.0016 (12)	−0.0007 (12)
P1	0.0199 (3)	0.0183 (4)	0.0216 (4)	−0.0015 (3)	0.0008 (3)	−0.0005 (3)
F1	0.0531 (12)	0.0476 (12)	0.0228 (11)	−0.0131 (10)	−0.0014 (9)	0.0103 (9)
F2	0.0484 (11)	0.0258 (10)	0.0345 (12)	0.0000 (9)	−0.0003 (9)	−0.0105 (9)
F3	0.0205 (8)	0.0374 (11)	0.0619 (15)	−0.0024 (8)	−0.0029 (9)	−0.0005 (11)
F4	0.0583 (12)	0.0407 (12)	0.0340 (13)	−0.0018 (10)	0.0028 (9)	0.0167 (10)
F5	0.0221 (8)	0.0325 (11)	0.0683 (15)	−0.0025 (8)	−0.0081 (9)	0.0000 (10)
F6	0.0387 (10)	0.0321 (11)	0.0560 (14)	−0.0106 (8)	0.0194 (10)	−0.0224 (10)

### Geometric parameters (Å, º)

**Table:**

Au1—N1	2.032 (2)	C6—C7	1.388 (4)
Au1—N2	2.036 (2)	C7—C10	1.406 (4)
Au1—Cl1	2.2506 (7)	C7—C8	1.440 (4)
Au1—Cl2	2.2549 (8)	C8—C9	1.348 (4)
N1—C1	1.325 (3)	C8—H8	0.9500
N1—C5	1.362 (4)	C9—H9	0.9500
N2—C12	1.322 (3)	C10—C11	1.370 (4)
N2—C6	1.372 (3)	C10—H10	0.9500
C1—C2	1.389 (4)	C11—C12	1.400 (4)
C1—H1	0.9500	C11—H11	0.9500
C2—C3	1.368 (4)	C12—H12	0.9500
C2—H2	0.9500	P1—F1	1.582 (2)
C3—C4	1.411 (4)	P1—F3	1.5877 (17)
C3—H3	0.9500	P1—F2	1.589 (2)
C4—C5	1.396 (4)	P1—F5	1.5976 (18)
C4—C9	1.430 (4)	P1—F4	1.602 (2)
C5—C6	1.414 (4)	P1—F6	1.6070 (19)
			
N1—Au1—N2	81.75 (9)	C10—C7—C8	124.8 (3)
N1—Au1—Cl1	174.84 (7)	C9—C8—C7	120.9 (3)
N2—Au1—Cl1	93.90 (6)	C9—C8—H8	119.5
N1—Au1—Cl2	95.11 (7)	C7—C8—H8	119.5
N2—Au1—Cl2	176.74 (6)	C8—C9—C4	122.0 (3)
Cl1—Au1—Cl2	89.28 (3)	C8—C9—H9	119.0
C1—N1—C5	120.1 (2)	C4—C9—H9	119.0
C1—N1—Au1	127.8 (2)	C11—C10—C7	119.7 (3)
C5—N1—Au1	112.00 (17)	C11—C10—H10	120.1
C12—N2—C6	120.2 (2)	C7—C10—H10	120.1
C12—N2—Au1	128.12 (19)	C10—C11—C12	120.2 (3)
C6—N2—Au1	111.69 (18)	C10—C11—H11	119.9
N1—C1—C2	121.0 (3)	C12—C11—H11	119.9
N1—C1—H1	119.5	N2—C12—C11	120.5 (3)
C2—C1—H1	119.5	N2—C12—H12	119.7
C3—C2—C1	120.4 (3)	C11—C12—H12	119.7
C3—C2—H2	119.8	F1—P1—F3	91.30 (11)
C1—C2—H2	119.8	F1—P1—F2	90.01 (11)
C2—C3—C4	119.5 (3)	F3—P1—F2	90.47 (10)
C2—C3—H3	120.3	F1—P1—F5	89.25 (11)
C4—C3—H3	120.3	F3—P1—F5	178.81 (11)
C5—C4—C3	117.2 (3)	F2—P1—F5	90.58 (11)
C5—C4—C9	117.5 (3)	F1—P1—F4	179.13 (12)
C3—C4—C9	125.3 (3)	F3—P1—F4	89.57 (11)
N1—C5—C4	121.9 (3)	F2—P1—F4	90.02 (11)
N1—C5—C6	117.3 (2)	F5—P1—F4	89.88 (11)
C4—C5—C6	120.8 (3)	F1—P1—F6	90.90 (11)
N2—C6—C7	121.9 (3)	F3—P1—F6	89.82 (10)
N2—C6—C5	117.0 (3)	F2—P1—F6	179.04 (12)
C7—C6—C5	121.0 (3)	F5—P1—F6	89.13 (10)
C6—C7—C10	117.4 (3)	F4—P1—F6	89.06 (12)
C6—C7—C8	117.8 (3)		
			
C5—N1—C1—C2	−1.0 (4)	C4—C5—C6—N2	178.2 (2)
Au1—N1—C1—C2	174.1 (2)	N1—C5—C6—C7	178.8 (2)
N1—C1—C2—C3	0.2 (5)	C4—C5—C6—C7	−1.4 (4)
C1—C2—C3—C4	1.0 (5)	N2—C6—C7—C10	2.1 (4)
C2—C3—C4—C5	−1.4 (4)	C5—C6—C7—C10	−178.4 (2)
C2—C3—C4—C9	178.5 (3)	N2—C6—C7—C8	−178.9 (2)
C1—N1—C5—C4	0.6 (4)	C5—C6—C7—C8	0.7 (4)
Au1—N1—C5—C4	−175.2 (2)	C6—C7—C8—C9	0.4 (4)
C1—N1—C5—C6	−179.7 (2)	C10—C7—C8—C9	179.4 (3)
Au1—N1—C5—C6	4.5 (3)	C7—C8—C9—C4	−0.8 (4)
C3—C4—C5—N1	0.6 (4)	C5—C4—C9—C8	0.1 (4)
C9—C4—C5—N1	−179.3 (3)	C3—C4—C9—C8	−179.8 (3)
C3—C4—C5—C6	−179.1 (2)	C6—C7—C10—C11	−0.7 (4)
C9—C4—C5—C6	1.0 (4)	C8—C7—C10—C11	−179.7 (3)
C12—N2—C6—C7	−2.3 (4)	C7—C10—C11—C12	−0.5 (4)
Au1—N2—C6—C7	177.4 (2)	C6—N2—C12—C11	1.0 (4)
C12—N2—C6—C5	178.2 (2)	Au1—N2—C12—C11	−178.6 (2)
Au1—N2—C6—C5	−2.2 (3)	C10—C11—C12—N2	0.4 (4)
N1—C5—C6—N2	−1.6 (3)		
